# Interrogating breast cancer heterogeneity using single and pooled circulating tumor cell analysis

**DOI:** 10.1038/s41523-022-00445-7

**Published:** 2022-07-05

**Authors:** Françoise Rothé, David Venet, Dieter Peeters, Ghizlane Rouas, Mattia Rediti, Dominiek Smeets, Floriane Dupont, Peter Campbell, Diether Lambrechts, Luc Dirix, Christos Sotiriou, Michail Ignatiadis

**Affiliations:** 1 Breast Cancer Translational Research Laboratory J.-C. Heuson, Université Libre de Bruxelles, Institut Jules Bordet, Brussels, Belgium; 2 Histopathology, Imaging and Quantification Unit, CellCarta, Antwerp, Belgium; 3 Department of Pathology, AZ Sint-Maarten, Mechelen, Belgium; 4 Laboratory of Translational Genetics, Department of Human Genetics, KU Leuven, Leuven, Belgium; 5 Center for Cancer Biology, VIB, Leuven, Belgium; 6 Wellcome Trust Sanger Institute, Hinxton, Cambridgeshire UK; 7 Translational Cancer Research Unit, Center for Oncological Research, University of Antwerp, Antwerpen, Belgium; 8 Department of Medical Oncology, Université Libre de Bruxelles, Institut Jules Bordet, Brussels, Belgium

**Keywords:** Breast cancer, Translational research, Tumour heterogeneity

## Abstract

Single cell technologies allow the interrogation of tumor heterogeneity, providing insights into tumor evolution and treatment resistance. To better understand whether circulating tumor cells (CTCs) could complement metastatic biopsies for tumor genomic profiling, we characterized 11 single CTCs and 10 pooled CTC samples at the mutational and copy number aberration (CNA) levels, and compared these results with matched synchronous tumor biopsies from 3 metastatic breast cancer patients with triple-negative (TNBC), HER2-positive and estrogen receptor-positive (ER+) tumors. Similar CNA profiles and the same patient-specific driver mutations were found in bulk tissue and CTCs for the HER2-positive and TNBC tumors, whereas different CNA profiles and driver mutations were identified for the ER+ tumor, which presented two distinct clones in CTCs defined by mutations in *ESR1* Y537N and *TP53*, respectively. Furthermore, de novo mutational signatures derived from CTCs described patient-specific biological processes. These data suggest that tumor tissue and CTCs provide complementary clinically relevant information to map tumor heterogeneity and tumor evolution.

## Introduction

Next-generation sequencing studies have demonstrated that cancer evolves over time and under the selective pressure of systemic treatment^1^. Temporal and spatial intratumor heterogeneity has now well been described in breast cancer (BC)^2,3^.

In the past years, the molecular assessment of circulating tumor cells (CTCs) hold the promise to become a valuable tool to map tumor heterogeneity and monitor tumor evolution^4–6^. In addition, the enumeration and characterization of CTCs offers several potential clinical applications, ranging from the early detection of cancer, the estimation of the risk of metastatic recurrence, the real-time monitoring of treatment efficacy as well as the identification of resistance mechanisms in different tumor types, including BC^6^. To date, CellSearch is the only Food and Drug Administration approved technology for the detection of CTCs, and together with the DEPArray system allows the isolation and genomic characterization of single CTCs^7–9^. Several technologies for enrichment, isolation and characterization of CTCs are currently under development and clinical validation^4,5,10^.

So far, few studies have used exome sequencing to analyze CTCs in BC and other tumor types^11–19^. Several studies have suggested that copy number aberrations (CNAs) profiles are usually consistent between CTCs and tumor tissue^13,17^, as well as among CTCs^18^. Using a targeted sequencing approach of 130 cancer-related genes, Paoletti et al. compared CTCs to tumor biopsies and detected at least one prioritized driver mutation in 85% of matched CTCs with higher discrepancy being observed for non-driver mutations^17^. Lohr et al. showed that mutations present in at least 3 CTCs were often present in the bulk tissue (70% of the cases) in two metastatic prostate cancer patients, while trunk mutations were found in 90% of the CTCs^12^. Furthermore, CTCs were found to mirror the clonal mutations of bone marrow tumor cells in multiple myeloma^15,16^. More recently, whole exome sequencing performed on 3 CTCs from one BC patient showed high genomic heterogeneity among the analyzed CTCs, with only few single-nucleotide variants (SNVs) shared by all CTCs^19^, whereas Su et al.^18^ showed that most of the mutations in the tumor tissue of small cell lung cancer patients were also present in CTCs. Importantly, the characterization of CTCs using whole genome sequencing may also guide treatment personalization^14^.

In this study, we investigated whether CTCs could complement metastatic biopsies for tumor genomic profiling allowing an optimized advanced stage BC patients’ care.

## Results

### Somatic mutations and copy number aberration landscape in CTCs from metastatic BC patients

We included in our study 11 single CTCs and 10 pooled CTC samples matched to 3 tumor biopsies as well as 3 pooled white blood cell (WBC) samples from 3 metastatic BC patients (Supplementary Table 1, Supplementary Fig. 1) representing the 3 major BC subtypes, namely triple-negative BC (TNBC) (patient #1), human epidermal growth factor receptor 2 (HER2)-positive/estrogen receptor (ER)-negative BC (patient #2) and ER-positive/HER2-negative BC (patient #3, luminal BC). Of note, time to metastatic relapse was 2.5 years and 8 years for patients with TNBC (#1) and ER+ (#3) tumors, respectively, whereas patient with HER2+ tumor (#2) presented de novo metastatic BC.

We interrogated the mutational landscape and copy number profiles of CTCs as well as matched bulk primary (#2) or metastatic (#1, #3) tumors. Tumor biopsies and CTC samples were taken synchronously. In the 21 evaluable CTC samples, we identified a median of 1395 high-confidence somatic SNVs (range = 203 to 3328 per sample) including a median of 573 nonsynonymous and nonsense SNVs (range = 75 to 1336). We also found a median of 105 CNAs (range = 32 to 149 per sample). In the 3 bulk tumors we identified 91, 144, and 323 high-confidence somatic SNVs including 32, 36, and 127 nonsynonymous and nonsense SNVs. We also found 80, 133, and 82 CNAs in the bulk tumor sample from patients with TNBC, HER2+ and ER+ tumors (#1, #2, and #3), respectively. Supplementary Table 2 provides information about the number of SNVs and CNAs identified for each patient.

### Comparison of SNVs between CTCs and synchronous bulk tumor tissue

**Fig. 1:**
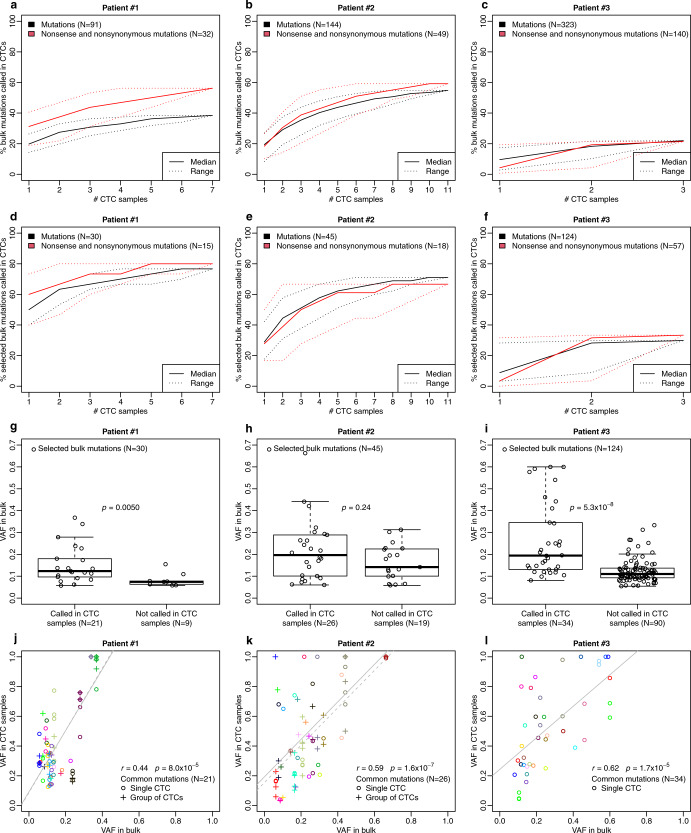
Comparison between single-nucleotide variation (SNVs) detected in bulk tumor and in CTCs. **a–c** Percentage of bulk mutations called in CTCs, in patient #1, #2, and #3, respectively. Mutations in bulk and CTCs were independently called using the Strelka algorithm. The solid lines are the median, the dotted lines the range. Black: All mutations. Red: Nonsense and nonsynonymous mutations. **d–f** Percentage of selected bulk mutations called in CTCs, in patient #1, #2 and #3, respectively. Selected bulk mutations are defined as bulk mutations with 20X coverage in at least one CTC. Mutations in bulk and CTCs were independently called using the Strelka algorithm. The solid lines are the median, the dotted lines the range. Black: All mutations. Red: Nonsense and nonsynonymous mutations. **g–i** Distribution of the VAFs of selected bulk mutations according to whether they are called or not in CTC samples. Selected bulk mutations are defined as bulk mutations with 20× coverage in at least one CTC and are represented as black circles. Mutations in bulk and CTCs were independently called using the Strelka algorithm. P-value (two-sided) compares the distribution of the VAFs of selected bulk mutations in the two groups (called vs. not called in CTCs) using a Wilcoxon test. **j–l** Spearman correlation (r) between VAF in bulk and VAF in CTCs, for selected bulk mutations called in CTC samples (common mutations), in patient #1, #2, and #3, respectively. Each color represents a specific mutation. Single CTC samples are represented as circles (o) and group of CTC samples as crosses (+).

**Fig. 2:**
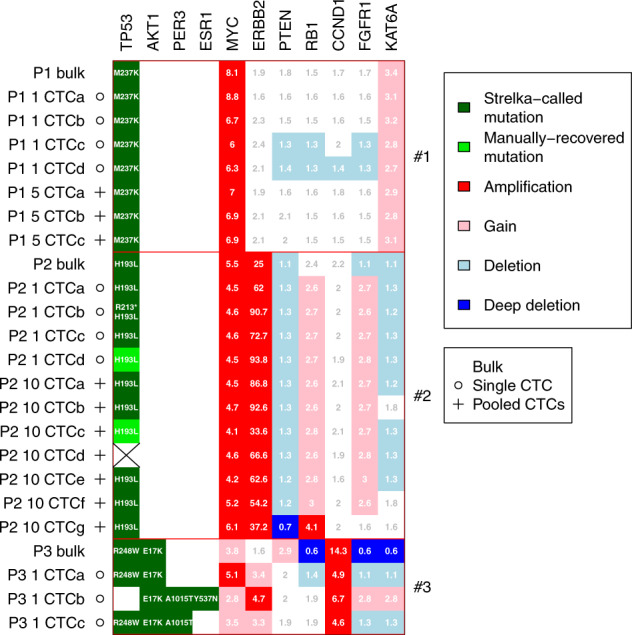
Driver mutations and copy number aberrations for each CTC and bulk sample for the three patients. Mutations were called using the Strelka algorithm (dark green). Mutations that were not called by Strelka but were manually recovered after visual inspection with at least one read showing the alternative allele are shown in light green. The stricken cell had no read covering the H193L mutation. Copy numbers are relative to a normalized ploidy of 2.

When comparing the mutational landscape of CTC samples to the matched, synchronous bulk tumor, we observed that 38% (TNBC patient #1), 55% (HER2+ patient #2), 22% (ER+ patient #3) of all bulk SNVs were found at least once in the CTC samples (Fig. 1a–c, Supplementary Tables 3–5). When focusing on selected bulk SNVs with adequate coverage on CTCs (at least 20 reads in the CTC samples), 79% (TNBC patient #1), 88% (HER2+ patient #2), 34% (ER+ patient #3) of all bulk SNVs were found at least once in the CTC samples (Fig. 1d–f; Supplementary Tables 3–5). When focusing only on SNVs in cancer driver genes, all SNVs identified in bulk were also identified in CTCs (Fig. 2). Of note, many SNVs that were not called in the CTC samples were actually present but were not called because of lack of sequencing depth or lack of read quality. In particular, the fractions of SNVs from the bulk that were covered with less than 5 reads in CTC samples were 60% (TNBC tumor #1), 64% (HER2+ tumor #2) and 41% (ER+ tumor #3). Interestingly, the percentage of bulk SNVs found in CTCs increased when considering nonsense/nonsynonymous SNVs only. Moreover, by increasing the number of CTC samples analyzed per patient, we recovered more SNVs in the CTC samples from the synchronous tumor tissue (Fig. 1a–f). Mutations present with a higher variant allele frequency (VAF) in the bulk tissue were detected more often in the CTC samples (Fig. 1g–i). Of note, low VAF mutations could be due to multiclonality, or having only one mutated allele in an amplified region, barring technical issues. However, as the VAFs in the bulk tumor were highly correlated with the VAFs in the CTC samples (Fig. 1j–l) multiclonality was not the predominant effect in our case.

**Fig. 3:**
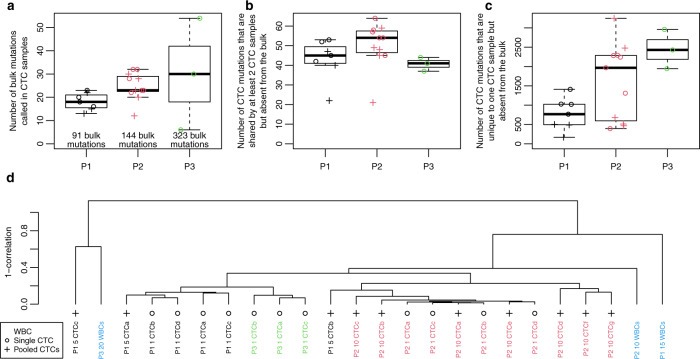
CTC mutation analysis. **a** Number of bulk mutations called in CTC samples in patients #1, #2 and #3, respectively. **b** Number of CTC shared mutations in patients #1, #2, and #3, respectively. These are mutations shared among at least 2 CTC samples but absent from the bulk. **c** Number of CTC private mutations present in only one CTC sample and absent in the bulk, in patients #1, #2 and #3, respectively. Mutations in bulk and CTCs were independently called using the Strelka algorithm. Black circles represent CTC samples. **d** Clustering of the CTCs based on the mutational pattern.

There were three types of mutations present in the CTC samples: 1. mutations that were also present in the bulk tumor (CTC bulk mutations, median = 23, range = 6 to 54), 2. mutations that were shared among at least 2 CTC samples and not present in the bulk (CTC shared mutations, median = 47, range = 21 to 64) and 3. mutations that were present in one CTC sample and absent in the bulk (CTC private mutations, median = 1309, range = 168 to 3250) (Fig. 3a–c and Supplementary Tables 6–8). It is difficult to assess for each mutation whether it is real or an artifact due to whole genome amplification^12^ or sequencing errors. Thus, the questions are whether a substantial proportion of those mutations is real, and if so, whether they could be clinically relevant. Since we found less than 100 private mutations on amplified WBCs, compared to 200 to 1500 on CTCs, this suggests that a large fraction of those mutations is not artifactual (Supplementary Tables 6–8). We reasoned that if those mutations were real, then they should be produced by processes that are to a certain extent patient-specific, and therefore should display a mutational pattern that is patient-specific as well. To verify this hypothesis, we calculated the number of mutations with specific context, as is done for mutational signature analysis, and used those to cluster the CTC and WBC samples (Fig. 3d). We found that CTC samples would mostly cluster by patient, with the exception of two samples from patient #1. The sample from the TNBC patient (#1) that did not fit the clustering at all (P1 5 CTCc) presented much less reads and mutations called (Supplementary Table 3), which may explain the discrepancy. The WBCs had few mutations which may explain why they did not cluster. Furthermore, there was no clear correlation between the mutational pattern of the WBCs, so it is not clear whether the mutations observed were real (e.g., due to ageing of immune stem cells)^20^ or technical artifacts.

### Comparison of CNA profiles between CTCs and synchronous bulk tumor tissue

**Fig. 4:**
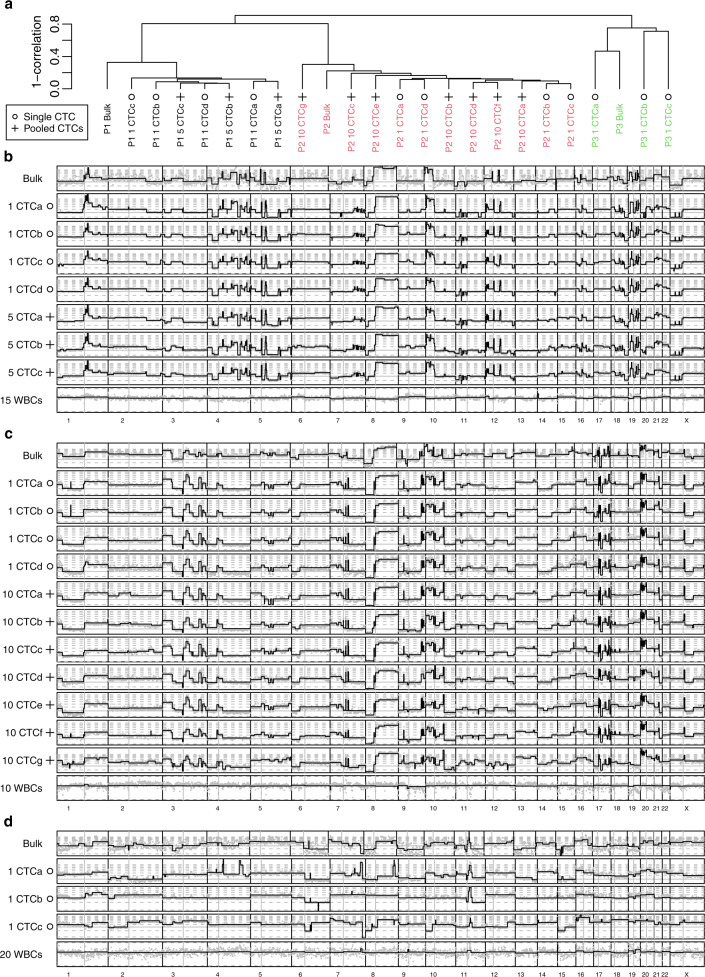
Copy number aberration analysis in the bulk tumor and in CTCs. **a** Clustering of all CTCs and bulk tumors, showing that samples cluster by patient. **b–d** Plot of the copy number by chromosome for all samples in patient #1, triple-negative (**b**), patient #2, HER2 positive (**c**) and patient #3 ER+/HER2− (**d**). Non-segmented estimates are given in light gray to show the estimate variance. ER Estrogen receptor, HER2 Human epidermal growth factor receptor 2.

We then compared the CNA profiles from the CTC samples and synchronous bulk tumor and found mostly concordant profiles for the patients with TNBC (#1) and HER2+ tumors (#2), with correlations of CNA profiles between CTC samples ranging from 63 to 94%. The correlations between CTC samples and bulk ranged from 62 to 72% for the patient with TNBC (#1) and 73 to 83% for the patient with HER2+ tumor (#2) (Fig. 4a–c). Conversely, the differences were much larger for the patient with ER+ tumor (#3), for whom we identified 3 different patterns (Fig. 4a, d): one CTC had a moderate correlation of 53% with the bulk, while all other CTC to CTC as well as CTC to bulk correlations ranged between 21 and 32%. In a clustering analysis, CTC samples of a given patient were grouped together as well as with the matched bulk tumor tissue (Fig. 4a).

The comparison of the CNA profiles showed that the evaluation for CTC samples was very reproducible, and less noisy than the corresponding profiles from bulk tissue (Fig. 4b–d). The noisier profiles for bulk tissues were due to the relatively low tumor purities (20 to 35% as determined by ABSOLUTE, Supplementary Table 9) which must be corrected for, and that amplify the random sampling variations. Of interest, CTC samples from the HER2+ BC (patient #2) did not show heterogeneity in terms of *ERBB2* copy number and were all HER2-amplified.

### SNVs and CNAs in known cancer driver genes

We identified SNVs and CNAs in known cancer driver genes including *TP53*, *AKT*, *MYC*, *PTEN*, and *CCND1* (Fig. 2). Importantly, we identified similar CNA aberrations and SNVs in driver genes in matched bulk and CTC samples, consistent with high-quality CTC isolation and sequencing (Fig. 4). In particular, we found *TP53* M237K mutation and *MYC* amplification in all samples from the TNBC patient (#1), *TP53* H193L mutation as well as *MYC* and *ERBB2* amplifications in all samples from the HER2+ patient (#2), *AKT* E17K mutation and *CCND1* amplification in all samples from the ER+ patient (#3). At the same time heterogeneity was observed between bulk and CTC samples or among CTC samples from the same patient. For HER2+ patient #2, we observed an *RB1* amplification in one CTC sample but not in the bulk. For ER+ patient #3, we observed an activating *ESR1* Y537N mutation and an *ERBB2* amplification in one CTC sample both absent in the bulk and the other CTC samples. Interestingly, this CTC sample was lacking a *TP53* mutation that was present in the bulk and the other CTC samples. An A1015T mutation in *PER3* (period circadian regulator) gene was observed in 2 CTC samples, while it was absent from the bulk and the other CTC sample. In this patient, a *PTEN* loss was also present in the bulk but absent from the CTC samples.

## Discussion

High-throughput molecular profiling studies have revolutionized our understanding of BC heterogeneity. It is well established now that BC evolves over time under the selection pressure of various factors including treatments received by the patients^2,3^. New studies are increasingly focusing on the characterization of the metastatic disease for treatment selection^21^. However, metastatic tissue biopsies can often be challenging to obtain. The molecular characterization of CTCs therefore appears as a promising approach that can provide unique insights into tumor temporal and spatial heterogeneity through a minimally invasive procedure. In this pilot study of 3 metastatic BC patients, we investigated whether profiling of CTCs at the single cell level can complement the synchronous metastatic bulk tumor tissue analyses.

We showed that CTCs were able to capture about 40% of SNVs identified in the bulk tumor analysis. This proportion increased when focusing on bulk mutations that were well covered on CTCs for the patients with TNBC and HER2+ BC, but not for the ER+ patient. For this patient time from diagnosis of metastatic disease to samples collection was significantly longer compared to the other 2 patients, and that might partly explain the differences observed. Of note, we also showed that by increasing the number of CTCs analyzed we can increase the percentage of identified SNVs from synchronous tumor tissue analyses. Moreover, SNVs with high VAF in tumor tissue were detected significantly more often on CTCs. The heterogeneity between CTCs and tumor tissue in terms of mutational profiles has been previously described, with a higher concordance reported for clonal mutations^12,15–18^.

Importantly, when focusing on bulk mutations in cancer driver genes, all of them were recovered using single CTC analysis. In addition, CTC analysis revealed mutations in cancer driver genes that were not identified in bulk analysis. Of particular interest, an activating mutation of *ESR1* in the ER+ patient, who previously progressed under endocrine therapy, was detectable only in one CTC and could not be identified by deep sequencing in the bulk tumor. The *ESR1* Y537N mutation has been associated with endocrine resistance and potential sensitivity to specific estrogen receptor degraders^22^, and has been previously described in CTCs^23^. Since this patient received endocrine therapy in both the adjuvant and metastatic settings before entering the study, this finding is not surprising. Interestingly, the CTC that had an *ESR1* mutation had no *TP53* mutation, contrary to the other CTCs and the bulk from the same patient. The exclusion between *ESR1* and *TP53* mutations has previously been reported for metastatic BC^24^.

Furthermore, we identified many SNVs in CTCs that could not be found in the tumor tissue. These SNVs likely represent passenger mutations with little clinical significance. This result has to be interpreted carefully, as whole genome amplification may introduce artifacts^12^. However, the number of private mutations in the WBCs samples was at least one order of magnitude lower as compared to the CTCs (Supplementary Table 6–8). Furthermore, by comparing *de novo* mutational signatures derived from WBCs and CTCs, we demonstrated that the distribution of the signatures was different between the patients, as well as between CTCs and WBCs. This supports the hypothesis that those mutations are not an artifact due to whole genome amplification and are produced by distinct biological processes that are mostly shared by the CTCs from the same patient (with the exception of the patient with ER+ tumor). Thus, mutational signatures can be used to identify the common origin and mutational process across CTCs with very different SNV profiles.

By comparing bulk tumor and CTC samples for CNAs, we showed that CTCs and paired bulk tissue clustered together. Furthermore, the CNA profiles of the CTCs in each patient showed overall high concordance, also when compared with the bulk tumor tissue for the TNBC and HER2+ but not for the ER+ patient, as observed in the SNV analyses. This finding confirmed previous studies in which CNA patterns were found to be reproducible across CTCs and compared to the tumor tissue^11,13,15–18^. For the patient with ER+ BC, as it was the case with SNVs, at the CNA level heterogeneity was observed between the bulk tumor and CTCs. Interestingly, in this patient, *ERBB2* copy number amplification was detected on a single CTC but not on bulk tumor analysis. Of note, the primary tumor diagnosed in 2005 presented a weak staining for HER2 by immunohistochemistry, while no staining was observed in the metastatic lesion. This exemplifies the power of single cell analysis to uncover subclonality. Further investigations aimed at evaluating the impact of *ERBB2* copy number heterogeneity detected in CTCs on anti-HER2 treatment response is warranted, even in patients with tumors classified as HER2-negative and especially in the light of the recent introduction of the “HER2-low” category^25^. Indeed, we and others have shown that HER2-positive CTCs can be detected in patients with HER2-negative tumors^26^. The recently reported DETECT study that randomized 105 patients with HER2-negative metastatic BC and HER2-positive CTCs between standard chemotherapy with or without lapatinib showed promising preliminary results of improved OS with the addition of lapatinib. Further validation in larger patient cohorts is needed.

Our study has some notable limitations. First, the small sample size, although representative of the 3 main BC subtypes, prevented us to perform additional analyses comparing CTCs characteristics across patients with the same BC subtype. This is an exploratory study that needs further validation using additional patients. Second, having characterized a single biopsy from one tumor site, we cannot exclude that the concordance in terms of SNVs could have been higher if multiple tissue samples were analyzed.

In conclusion, we showed that single CTC genomic analysis provides most of the information provided by the genomic analysis of the synchronous bulk metastatic tumors at least in terms of driver SNVs and CNAs. It additionally allows the identification of some driver aberrations that are not detected in the bulk and also provides information on the clonal/subclonal distribution of all aberrations identified. This information is not captured by ctDNA analysis, which does not inform us on the distribution of genomic aberrations in the different clones within the same patient. However, ctDNA analysis is easier to perform in the context of a clinical setting as compared to the tedious procedure of single CTC analysis, hence it remains the preferred liquid biopsy analyte tested in clinical trials and clinical practice. Indeed, multigene assays evaluating ctDNA are already approved for use in clinical practice, but it is not the case for CTCs. Nevertheless, we believe it is worth evaluating whether CTC heterogeneity provides clinically relevant information. The clinical value of this additional information provided by single CTC analysis including information on clonality needs to be prospectively evaluated in larger cohorts.

These data suggest that tumor tissue and single CTC exome sequencing analyses provide complementary information to map tumor heterogeneity and monitor tumor evolution. Further validation for potential clinical applications is needed.

## Methods

### Patients and samples

Three patients with metastatic BC treated at the Institut Jules Bordet were included in the study, one for each of the 3 major BC subtypes. For each patient, whole blood samples were collected simultaneously with the tissue biopsies. Samples’ collection was obtained after several lines of treatment for metastatic disease for patients #1 and #3, and at the time of diagnosis for de novo metastatic disease in patient #2. Detailed characteristics of the patients included in the study are provided in Supplementary Table 1. The study was approved by the Institut Jules Bordet ethics committee (internal number 1698) and was conducted in accordance with the Declaration of Helsinki, written informed consent being obtained from all participants.

### Circulating tumor cell isolation

CTCs were enumerated and enriched from whole blood samples using the CellSearch system, according to the manufacturer’s recommendations (Janssens Diagnostics, LLC). CellSearch cartridges were processed to recover single and pools of CTCs using the DEPArray^TM^ technology (Menarini Silicon Biosystems). WBCs were isolated using the same procedure as the CTCs. Whole genome amplification (WGA) of the DNA from single and pools of CTCs and WBCs was performed using the Ampli1 kit (Menarini Silicon Biosystems), as previously described^9^. Quality control of the WGA was assessed using the Ampli1 QC kit.

### Whole exome sequencing

DNA extraction was performed using the QIAamp DNA FFPE Tissue kit (Qiagen) and the DNeasy Blood and Tissue kit for the FFPE tumor tissues and whole blood samples respectively. Library preparation was performed using the Truseq Exome kit (Illumina). Whole exome library sequencing was performed using the Illumina HiSeq2000 platform with a 200× targeted coverage. Eleven single CTCs (4, 4 and 3 for patients #1, #2 and #3, respectively) and 10 pooled CTC samples (3 and 7 for patients #1 and #2, respectively) comprising 5 (patient #1) or 10 (patient #2) CTCs were sequenced (Supplementary Tables 6–8), for a total of 21 CTC samples, as well as 3 pools of WBCs (one for each patient).

### Mutation analysis

Reads were trimmed using Trimmomatic (version 0.36)^27^ and aligned using bwa (version 0.7.17)^28^. Duplicates were removed using Picard tools (version 2.17)^29^. Variants were called with Strelka (version 2.9.2)^30^, using the whole blood as the normal. Mutations fitting the pattern TTAACTGACAGC were considered as artifacts and removed, as were mutations appearing in more than one patient. Mutations with a VAF below 20% were discarded, unless they were present in the corresponding bulk sample.

Bulk mutations were defined as mutations present in the bulk but absent from both whole blood normal DNA and amplified WBC from the same patient. CTC mutations were defined as mutations present in at least one CTC sample but absent from the WBC cells sample from the same patient. CTC shared mutations were defined as CTC mutations that are shared by at least 2 CTC samples but absent from the bulk. CTC private mutations were defined as CTC mutations that were unique to only one CTC sample but absent from the bulk.

Frequencies of mutations by context (flanking bases) were obtained with the R package deconstructSig^31^. Hierarchical clustering on those frequencies was done with complete linkage, using 1-cor(log(0.01 + f)) as the distance.

We selected to show on the Oncoplot genes that had at least 2 mutations across all samples that appeared at least 20 times in COSMIC, in addition to *ESR1* and selected driver CNAs. Individual mutations shown appear at least 20 times in COSMIC, except for *TP53* mutations for which the cutoff was set at 5 appearances in COSMIC.

### Copy number assessment

CNAs were determined by counting reads in 10 kb windows. Those counts were normalized using WBC pools (for CTC/CTC pools) or normal blood (for bulk tumor). Segmentation was performed using circular binary segmentation from the DNAcopy^32^ R package. Correction for purity for bulk tumor sample was performed with ABSOLUTE^33^. Pairwise concordance of CNAs profiles was assessed using Spearman correlation of CNAs profiles across the whole genome. Using copy numbers normalized to a sample ploidy of 2, amplifications were defined as CN > 4, gains as CN > 2.5, deletions as CN < 1.5 and deep deletions as CN < 0.8.

### Statistical analyses

Comparisons between groups for continuous variables (i.e., distribution of the VAFs of selected bulk mutations in two groups, called vs. not called in CTCs) were performed using a Wilcoxon test. *P* values were two-sided, no correction for multiple testing was performed. Correlations were Spearman, *p* values on correlations were obtained with the cor.test R function. Boxplots were standard R boxplots, so center lines are at the median, hinges are at the first and third quartiles, while the whiskers extend to the most extreme data point which is no more than 1.5 times the interquartile range from the box. Analyses were performed using the R software (version 3.5).

## Supplementary information

Supplementary Information

## Data Availability

The whole exome sequencing data from this publication have been deposited to the EGA database (https://ega-archive.org) and assigned the identifier EGAS00001005228.

## Code availability

Open-source software were used for this study. The scripts used are available upon request.
