# Supplementary material for: Interrogating breast cancer heterogeneity using single and pooled circulating tumor cell analysis
Source: NPJ Breast Cancer. 2022 Jul 5;8:79. doi: 10.1038/s41523-022-00445-7 (PMC9256697; doi:10.1038/s41523-022-00445-7)
Supplement: Supplementary file 1 — Supplementary Information [file 41523_2022_445_MOESM1_ESM.pdf]

Supplementary Figure 1. Timeline of the 3 patients.

Timing of the main tumor-related events are shown, as well as the timing of biopsies and CTC samples collection.

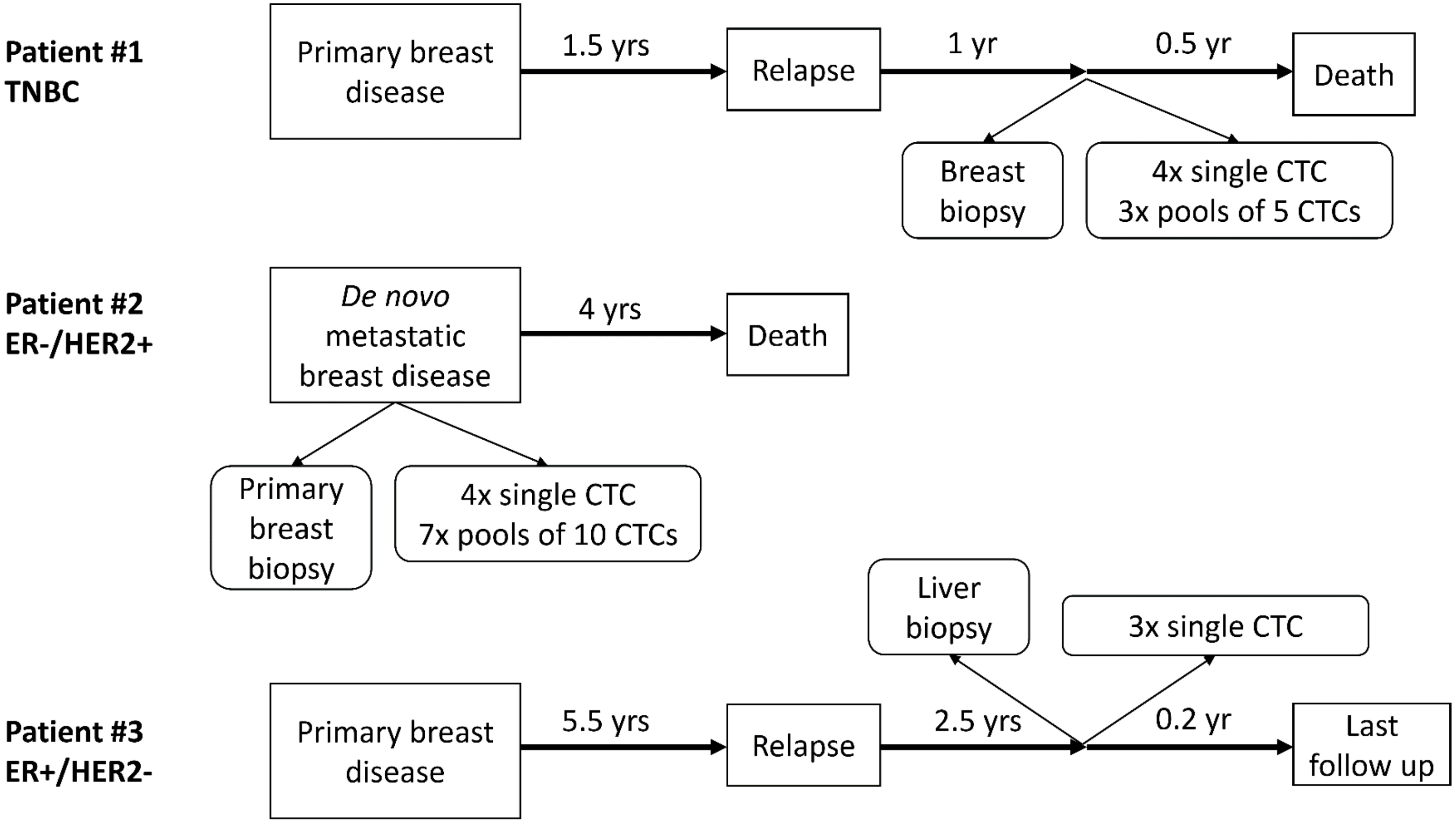

Supplementary Table 1. Patient and tumor characteristics from the 3 patients included in the study.

					Primary tumor characteristics					Biopsy characteristics							Lines of treatment received before study inclusion		
**ID**	**BC type**	**Time to relapse**	**Age at diagnosis**	**Metastatic sites at study entry**	**histology**	**grade**	**ER**	**PR**	**HER2**	**organ**	**histology**	**grade**	**ER**	**PR**	**HER2**	**Ki67**	**CHT**	**HT**	**Targeted Agent**
#1	Triple negative	2.5 years	54	lymph node, pleura, liver, skin	ductal	2	ER-	PR-	HER2-	breast	ductal	2	ER-	PR-	HER2-	90%	3	0	1
#2	ER-/HER2+	de novo metastatic	49	lymph node, bone, lung, liver, skin, muscle, eye	ductal	2	ER-	PR-	HER2+	primary breast	ductal	3	ER-	PR-	HER2+	60%	0	0	0
#3	ER+/HER2-	8 years	51	liver, bone	mixed	NA	ER+	PR+	HER2-	liver	NA	NA	ER-	PR-	HER2-	<10%	3	2	1

BC = breast cancer

ER = estrogen receptor

HER2 = human epidermal growth factor receptor 2

PR = progesterone receptor

CHT = chemotherapy

HT = hormonotherapy

Supplementary Table 2. SNVs and CNAs identified in bulk tumor as well as CTC samples. Median and range values are provided for each patient.

**Patient ID**	**TNBC #1**	**HER2+ #2**	**ER+ #3**
**SNV in CTC samples**	831 (203 to 1477)	2041 (475 to 3328)	2527 (2011 to 3006)
**nonsynonymous/nonsense SNV in CTC samples**	401 (80 to 655)	935 (207 to 1463)	1101 (910 to 1341)
**SNV in bulk samples**	91	144	323
**nonsynonymous/nonsense SNV in bulk samples**	32	49	140
**CNA in CTC samples**	187 (174 to 221)	127 (115 to 166)	97 (62 to 98)
**CNA in bulk samples**	80	113	82

Supplementary Table 3. Somatic SNVs identified in the CTC samples from patient with TNBC (#1).

chrom	pos	ref	alt	gene	Nref	Nalt	VAF	1 CTCa	1 CTCb	1 CTCc	1 CTCd	5 CTCa	5 CTCb	5 CTCc
chr1	7980899	A	T	TNFRSF9	204	25	0,1092	1/2	**2/2**	2/2		1/1		
chr1	44437288	C	G	DPH2	85	6	0,0659							
chr1	109642878	G	C		30	6	0,1667	**7/7**		3/3	1/1	2/5	**5/5**	
chr1	118497291	G	C		43	5	0,1042	1/4	1/4	0/5	0/1	1/5	0/1	0/1
chr1	152193276	A	G	HRNR	122	15	0,1095					0/30		
chr1	159832239	C	T		86	101	0,5401	**12/13**	**6/8**	**12/15**	**8/10**	**12/13**	**6/7**	**4/4**
chr1	167408587	G	C	CD247	97	8	0,0762	**14/43**	0/36	**9/55**	1/38	**9/27**	**13/21**	**3/17**
chr2	54872338	A	G		38	6	0,1364	0/25	**7/27**	6/27	5/26	1/18	**6/19**	1/3
chr2	79313419	C	T		50	9	0,1525							
chr2	89878487	G	C		61	5	0,0758	0/11	0/7	0/3	0/8	0/22	0/4	0/2
chr2	179459236	C	T	TTN	73	6	0,0759	**6/6**	0/3				1/1	
chr3	49568307	C	A	DAG1	67	9	0,1184		2/3	0/3		2/3	1/3	
chr3	113377662	G	C	USF3	169	17	0,0914	0/67	**14/27**	**8/22**	0/10	2/22	**17/40**	**9/18**
chr3	126679043	G	A	CHCHD6	76	12	0,1364							
chr3	152847195	G	A		22	4	0,1538	0/1	0/2	0/17	0/17	0/3	0/6	0/5
chr4	49139234	A	T		94	8	0,0784	1/5	0/2	1/7	0/2	0/8	1/5	0/1
chr4	94085961	C	T		52	5	0,0877		0/1		0/1			
chr4	108935771	T	C		156	11	0,0659	0/41	1/40	0/42	1/17	1/29	0/21	0/20
chr4	115544795	C	T	UGT8	129	75	0,3676	**89/114**	**51/51**	**56/57**	**46/46**	**45/45**	**82/89**	**28/28**
chr4	126238020	C	T	FAT4	249	20	0,0743	0/4	2/13	**5/17**	2/16	2/22	**8/18**	0/3
chr5	191809	G	A	LRRC14B	39	7	0,1522							
chr5	24749483	A	G		25	5	0,1667	0/4	0/5	0/4		0/2	0/3	0/2
chr5	127623040	C	T	FBN2	78	9	0,1034	**4/33**	**16/44**	**12/30**	**14/41**	**11/37**	**13/47**	**9/15**
chr6	54186214	T	A		65	5	0,0714	0/15	2/8	2/6	3/7	2/6	2/11	0/6
chr6	88075011	C	A	C6orf163	58	9	0,1343	1/40	**12/40**	**9/40**	**8/25**	3/16	2/26	2/18
chr6	99893989	C	G	USP45	106	7	0,0619	0/25	**5/16**	**7/26**	**9/13**	2/13	0/14	2/6
chr6	125379084	A	G		65	9	0,1216			0/1			0/1	
chr8	55533891	G	A	RP1	146	9	0,0581	**6/9**	2/6	0/20	1/3	2/9		0/1
chr8	101078454	G	A	RGS22	85	10	0,1053	1/9	1/7	**6/11**	1/7	2/5	0/10	0/10
chr9	66455393	C	T		29	6	0,1714							
chr9	68409680	C	G		41	6	0,1277			0/1				0/1
chr9	139996438	A	C	MAN1B1	201	17	0,078	0/102	0/145	0/219	0/153	0/137	1/72	0/16

chr10	42389587	G	A		129	9	0,0652			1/1				
chr11	51580489	T	G		157	9	0,0542							
chr11	55136144	G	C	OR4A15	112	12	0,0968	**14/19**	**12/21**	**8/21**	**8/14**	5/8	**9/16**	1/3
chr11	67207261	C	T	CORO1B	58	12	0,1714		1/1	0/3	1/1	1/1		
chr12	3392390	C	G		35	5	0,125				1/1		0/1	
chr12	6422182	G	C		63	8	0,1127					0/1		
chr12	8289361	G	C		206	19	0,0844	3/11	**3/15**	1/13	1/9	**3/9**	**7/23**	1/15
chr12	8289411	G	C	CLEC4A	237	30	0,1124	**4/32**	**4/25**	**14/42**	**8/27**	**7/22**	**16/54**	**5/24**
chr12	8289471	G	C	CLEC4A	234	33	0,1236	3/30	**3/22**	**17/43**	**6/23**	**7/15**	**18/56**	**4/15**
chr12	8289489	G	A	CLEC4A	234	31	0,117	3/29	**3/22**	**13/37**	**4/22**	**7/17**	**17/53**	2/14
chr12	8289546	G	C		141	19	0,1188	0/16	0/6	**6/18**	**3/11**	**4/12**	**5/24**	0/12
chr12	21531170	G	A		182	11	0,057	**15/58**	**25/54**	**11/39**	**11/35**	**6/21**	**13/40**	1/12
chr14	96001712	C	T	GLRX5	36	7	0,1628							
chr14	102472314	G	A	DYNC1H1	71	17	0,1932	0/1		0/1	1/2	1/1	0/1	2/3
chr14	105945497	C	T	CRIP2	67	5	0,0694							
chr15	31776672	C	T	OTUD7A	147	18	0,1091					0/6		
chr16	33973718	A	G		38	8	0,1739	3/55	4/48	6/43	3/32	3/56	3/53	**10/35**
chr16	46391488	G	A		270	17	0,0592							
chr16	57741613	G	A		91	14	0,1333							
chr17	7577571	A	T	TP53	41	21	0,3387	**35/35**	**19/19**	**27/27**	**29/29**	**43/43**	**27/27**	**9/9**
chr17	42462514	G	T		46	12	0,2069					2/2		
chr17	48824049	A	T	LUC7L3	108	15	0,122		**7/7**	0/3		2/9	**5/5**	1/4
chr17	53068122	T	A		27	7	0,2059							
chr17	60744671	A	G		52	6	0,1034		1/1	1/2		0/1		2/2
chr18	18512329	G	A		60	11	0,1549	7/51	5/85	13/70	7/54	8/67	6/63	4/79
chr19	7626553	C	T		38	9	0,1915	1/2		1/1		2/3	0/1	1/1
chr19	7684568	C	A		67	5	0,0694							
chr19	7831588	A	T	CLEC4M	163	11	0,0632	0/2	2/10		0/6	3/34	1/5	
chr19	12876914	C	T		58	6	0,0938				1/1		0/1	
chr19	12878357	C	G		73	8	0,0988	2/3	0/1	0/3	0/1			0/2
chr19	15341999	C	T		56	5	0,082						0/5	
chr19	17305841	C	G	MYO9B	99	9	0,0833	0/2	**2/2**	0/1	0/2	0/11		
chr19	33587261	A	G	GPATCH1	105	16	0,1322	0/1	1/2	1/4	**3/4**	0/3	2/11	1/2
chr19	36435716	G	A	LRFN3	33	8	0,1951							
chr21	26734749	C	G		44	17	0,2787	**17/36**	**33/43**	**29/44**	**20/30**	**30/36**	**30/38**	**10/15**

chr21	30250584	A	G	N6AMT1	75	6	0,0741	2/2	0/1	1/2		0/2	1/2	1/2
chr21	34927158	G	A	SON	72	42	0,3684						1/3	
chr21	45733555	C	T	PFKL	58	6	0,0938					1/3	0/1	
chr22	24086357	A	C		97	13	0,1182	2/9	1/3	1/7		1/18		
chr22	44287155	G	A	PNPLA5	19	5	0,2083							
chr22	46654589	A	T	PKDREJ	69	11	0,1375	**19/26**	**13/21**	**17/58**	**16/28**	**19/50**	**15/30**	**2/6**
chrX	82763896	G	A	POU3F4	70	10	0,125			1/1				
chrX	133379486	A	G	CCDC160	132	9	0,0638			2/3			0/2	

chrom: chromosome
pos: position of the chromosome
ref: reference allele
alt: alternative allele
VAF: variant allele frequency in the bulk sample

Supplementary Table 4. Somatic SNVs identified in the CTC samples from patient with HER2+ tumor (#2).

**chrom**	**pos**	**ref**	**alt**	**gene**	**Nref**	**Nalt**	**VAF**	**1 CTCa**	**10 CTCa**	**1 CTCb**	**10 CTCb**	**1 CTCc**	**10 CTCc**	**10 CTCd**	**10 CTCe**	**1 CTCd**	**10 CTCf**	**10 CTCg**
chr1	1685590	G	A	NADK	48	33	0,40741		**10/10**	**5/5**	0/15	**10/10**		1/1			0/2	
chr1	6529245	C	G	PLEKHG5	57	6	0,09524	0/1	**5/5**		**2/2**	1/1			0/1	0/1	2/2	
chr1	19494624	G	T	UBR4	57	8	0,12308	0/8	0/33	0/5	0/2		0/2		0/8		0/4	0/1
chr1	27429723	G	T	SLC9A1	72	20	0,21739	**3/9**			**10/11**	**6/6**	1/1		**5/7**	1/1	**5/7**	0/4
chr1	32670240	C	A	CCDC28B	21	6	0,22222							0/1	0/2			
chr1	32790076	C	G		24	7	0,22581	0/8		0/11	0/7	0/11	**15/27**	**8/9**	0/14		1/13	0/6
chr1	33291746	C	G	S100PBP	31	14	0,31111	0/9		**12/12**	**3/4**		0/3	0/5	0/8	0/2	**4/9**	
chr1	45115323	C	T		53	13	0,19697				0/2	0/50	0/3			0/8	0/1	
chr1	156018385	C	G	UBQLN4	90	13	0,12621		0/1	1/3	0/1	0/1	0/3		**1/1**	0/2	0/1	
chr1	156714020	C	G	HDGF	188	28	0,12963	0/1			3/3		0/1		0/28	1/18	2/7	
chr1	158746676	G	A	OR6N2	141	11	0,07237	0/1	0/4	0/4	0/2	0/2			0/11	0/4	0/6	0/2
chr1	177917076	C	T	SEC16B	34	8	0,19048	0/1	1/2	0/1	0/1	**5/9**	1/3	0/1	4/14	**3/9**	0/4	1/1
chr1	181720950	C	T		35	12	0,25532	1/2	1/1	0/1		0/3	2/4				1/2	1/3
chr1	204945625	C	T		90	6	0,0625	0/8	0/3	0/1	0/4		0/2	0/2	0/2	0/3	0/1	0/1
chr1	205499635	G	C		27	7	0,20588	0/1			0/7					1/3	0/2	
chr1	222832114	C	G	MIA3	146	35	0,19337	0/2	0/7		**4/5**	0/1	0/12			0/1	0/4	**4/4**
chr1	229654442	T	C		25	5	0,16667	0/1	0/2	0/1		0/4	0/3		0/1	0/4	0/3	0/1
chr1	237431083	T	G		74	21	0,22105				0/1		0/1				0/1	
chr2	69601131	A	G		56	6	0,09677											
chr2	84904177	C	A	DNAH6	38	13	0,2549	2/14	0/8	**4/5**	1/3	1/3	**4/7**	1/1	**13/14**	3/13	0/4	1/5
chr2	85892841	G	A	SFTPB	37	18	0,32727		**3/6**		3/6		1/2				**5/6**	2/3
chr2	133014557	G	A		85	6	0,06593											
chr2	162567158	G	A		37	6	0,13953	0/10	0/4	**5/5**	0/4	1/6	2/6	3/5	0/4	0/5	0/1	0/4
chr2	216249677	C	T	FN1	46	8	0,14815	0/4	0/5	0/2	0/7	0/13	0/1	0/3	0/10	0/2	0/2	0/3
chr2	219339380	C	T	USP37	139	11	0,07333	**4/10**			**3/5**	0/6	1/9	2/9		4/10	3/10	0/9
chr2	219532696	T	C	RNF25	22	8	0,26667	**3/8**	0/1	0/1	1/1	1/18	2/4		**4/5**	0/5	0/2	1/3
chr3	12859257	C	G	CAND2	115	9	0,07258	0/2	0/13	0/3	0/2		0/8		**4/6**	0/3	0/1	
chr3	15071821	C	T	NR2C2	230	14	0,05738	2/10	2/3	1/4	1/2	0/4	1/3	1/9	0/8	**5/8**	0/8	1/6
chr3	15106620	T	A	MRPS25	90	39	0,30233	5/11	1/1		**15/19**	0/21	3/15	3/11		**3/3**	0/6	
chr3	15529846	C	T		33	8	0,19512		0/2		0/4	1/27			0/7	0/8		0/1
chr3	52431063	G	C		56	22	0,28205					**7/7**						1/1

chr3	75788109	G	A	ZNF717	8	5	0,38462											
chr3	113379095	G	A	USF3	151	43	0,22165	0/3	0/3	0/5	0/3	0/5	0/5	0/2	0/5	0/7	0/9	0/5
chr3	126364993	C	G		117	32	0,21477	0/6	0/5		0/3		**4/12**	2/5	3/12	1/7	2/2	**3/6**
chr3	196434790	G	T		84	9	0,09677	**2/2**	0/3	**3/5**	0/4	**4/4**	**4/4**		0/1	2/3	0/4	0/1
chr4	6623071	G	A		81	7	0,07955	0/3			0/3		0/1				0/1	
chr4	49123058	G	C		53	5	0,08621	0/29	1/24	1/87	**6/105**	**6/141**	4/117	0/6	1/44	0/37	**3/159**	1/76
chr4	49156527	C	T		113	10	0,0813	1/4	0/2	**0/8**	**0/14**	0/10	0/7		0/3	0/1	**0/16**	0/8
chr4	54231440	G	A	SCFD2	110	19	0,14729	0/6	0/3	0/1	**3/4**		0/5	0/3		0/2	0/7	0/8
chr5	1258879	A	C		39	20	0,33898		0/1						0/2			0/2
chr5	32209744	A	T		12	4	0,25											
chr5	80655770	G	T	ACOT12	60	6	0,09091											0/1
chr5	134113427	C	G		43	11	0,2037											
chr5	140596019	C	A	PCDHB13	132	10	0,07042				0/18							
chr5	148586510	G	A		35	8	0,18605	**3/8**	0/5		2/12	**4/19**	3/9	2/4	0/13	1/21	**13/28**	**6/10**
chr5	176523196	C	T		39	12	0,23529											
chr5	179287282	T	C		48	4	0,07692			0/1			0/1	0/3	0/1	0/1	0/3	0/2
chr6	31737509	G	A	VWA7	39	7	0,15217	**8/8**	1/10	**8/15**	0/5		**11/12**	2/5	0/15	**9/18**	0/5	1/2
chr6	75884874	C	G	COL12A1	89	9	0,09184	1/3	**4/12**	**5/15**	1/3	**4/10**	1/8	**2/2**	**3/6**	3/16	1/7	0/9
chr6	117687199	G	A		39	10	0,20408						0/1		1/1			
chr6	127768828	C	T	SOGA3	53	22	0,29333	**9/31**	0/12	**3/6**	**5/7**	**6/14**	**9/14**	**5/6**	5/19	**16/16**	3/10	0/1
chr7	22197406	C	A		87	7	0,07447	0/4	**5/9**	0/5	1/3	2/6	0/5	2/7	2/8	**3/8**	**3/14**	**3/4**
chr7	61979304	A	G		39	6	0,13333					0/1	0/3	0/1				
chr7	99507268	A	T	TRIM4	90	41	0,31298	0/10	0/9	11/26	17/19	28/48	6/6	7/12	0/16	15/15	10/21	4/16
chr7	150937674	T	A		33	7	0,175	0/6	0/1	0/2	0/1		0/1	0/2	0/5	0/2	0/3	0/2
chr7	158334233	G	C	PTPRN2	35	6	0,14634	**17/17**	**5/11**	0/17	0/13	**5/5**	3/12		**9/10**	**5/5**	2/7	1/1
chr8	417612	T	A		31	11	0,2619											
chr8	31497520	C	G	NRG1	16	8	0,33333											
chr8	72123392	T	A	EYA1	173	12	0,06486	0/51	0/23	0/67	0/36	0/30	0/50	0/18	0/57	0/30	0/30	0/43
chr9	17795576	G	A	SH3GL2	25	12	0,32432					1/1	**2/2**					1/1
chr9	101900236	T	C	TGFBR1	127	21	0,14189	0/9	0/9	0/7	0/5	0/10	0/10	0/4	0/21		0/6	0/11
chr9	139379174	G	T	C9orf163	108	9	0,07692											
chr10	860867	G	A		76	26	0,2549	**2/4**	0/2	0/5	1/1	0/26	**4/5**	**2/2**	0/6	0/21	**5/10**	2/5
chr10	7749070	A	G		137	9	0,06164	1/13	1/24	3/14	0/15	2/25	4/36	0/5	0/8	1/14	1/6	1/3
chr10	12257724	C	T		231	20	0,07968	0/14	0/9	0/9	0/16	0/12	0/14	0/11	0/13	0/9	0/10	0/9
chr10	12257804	C	A	CDC123	258	17	0,06182	0/7	0/6	0/6	0/9	0/8	0/7	0/6	0/6	0/7	0/7	0/5

chr10	42392282	T	A		162	10	0,05814			0/4	0/5	0/9	0/3			0/1	0/4	0/4
chr10	42681020	G	T		97	12	0,11009											
chr10	43606776	C	T	RET	196	26	0,11712			1/1	0/1							
chr10	46999502	A	T		78	10	0,11364			0/2		0/4	0/1					
chr10	71662600	A	G	COL13A1	33	5	0,13158	**3/3**	3/21	0/5	0/2	0/1			0/1		0/4	0/1
chr10	73862008	T	C		57	45	0,44118	**19/25**	**5/8**	**22/22**	**13/14**	**17/25**	**17/18**	**4/5**	**20/20**	**42/47**	**12/25**	**25/30**
chr10	87676004	G	C		70	6	0,07895	0/1	0/2		0/10				0/1			
chr10	104176352	C	T	PSD	29	9	0,23684			1/1		1/1		2/2	**12/12**	**3/3**		
chr11	5264313	G	T		90	7	0,07216	**7/18**	0/20	0/21	**10/11**	3/33	**16/20**		0/10	4/9	3/8	1/15
chr11	61133485	T	G		33	24	0,42105	**17/35**	**18/18**	**2/2**	**6/14**	**13/13**	**14/16**	1/2	2/2	**36/40**	**9/12**	
chr11	76507090	C	G		216	19	0,08085	0/9	0/8	0/2	0/12	0/5	0/3			0/12	0/1	0/2
chr12	27849886	C	A	REP15	114	14	0,10938	**6/8**	**5/8**	0/4	2/4		2/4	**3/3**	0/4	0/1	2/3	**5/5**
chr12	46320859	G	A	SCAF11	69	16	0,18824	0/1	0/6		0/3	0/1			0/3			
chr12	57538956	G	A		61	23	0,27381			**2/2**	3/4	0/18		0/2		1/9	1/3	0/1
chr12	88514928	G	C	CEP290	35	9	0,20455	0/2	0/4	0/3	0/2	0/1	0/6	0/4	1/5	0/6	0/4	1/4
chr12	101584273	G	C	SLC5A8	68	38	0,35849	2/3	2/3		0/3		2/3			0/2	2/3	0/1
chr12	121131974	G	A	MLEC	21	10	0,32258	**14/35**	2/2	0/6	3/23	**14/14**	**14/37**	**3/8**	**19/33**	0/4	**5/15**	0/7
chr13	23411244	A	G		65	7	0,09722		0/1	0/1	0/1	1/1	1/1	0/1	0/1	0/1	0/1	0/1
chr13	46425723	G	A	SIAH3	67	29	0,30208		3/85	**7/29**	0/5	0/5	3/5	1/1	0/17	0/41	0/14	**4/15**
chr13	73636171	A	T	KLF5	126	45	0,26316	0/9		**27/33**	**20/30**	**12/17**	1/3	3/4	1/1		1/1	
chr14	38091506	T	A		92	29	0,23967				0/2	0/3	0/2	0/2	0/6		0/3	
chr14	60212452	G	C	RTN1	32	13	0,28889	**27/27**	0/14	**8/8**	0/24	**29/29**	**17/38**	**3/7**	0/24		**12/27**	**6/6**
chr14	105412633	G	A	AHNAK2	60	7	0,10448			0/3		0/1						0/2
chr15	43903077	G	A		142	9	0,0596											
chr15	58984781	G	A	HSP90AB4P	42	11	0,20755	**25/84**	0/36	0/31	**20/50**	**13/51**	**33/54**	**4/20**	0/56	**14/55**	**9/28**	**9/25**
chr15	75819553	T	C	PTPN9	106	44	0,29333	1/1	0/5	1/1	0/4	**4/7**	1/3	1/1	1/2	0/3	0/2	0/1
chr16	1712735	C	T		96	16	0,14286	0/3							**9/26**	**8/20**	0/1	
chr16	7703800	C	G		32	18	0,36	1/2	0/1								1/1	
chr16	15711282	C	T	MARF1	28	9	0,24324	**8/41**	0/38	0/32	**14/35**	0/10	3/33		0/1	0/25	4/7	1/8
chr16	15714299	C	A		113	7	0,05833					1/1		0/4	0/3			0/1
chr16	46402784	T	C		70	9	0,11392											
chr17	4843158	G	A	SLC25A11	188	12	0,06				0/9	0/42	0/1		**32/32**			0/1
chr17	7350144	T	G		18	5	0,21739	0/2		0/1	2/6	**24/24**		0/2		**2/2**	0/6	0/4
chr17	7578271	T	A	TP53	29	57	0,66279	**15/15**	**31/31**	**36/36**	**10/10**	**100/100**	15/16		**8/8**	2/2	**15/15**	**5/5**
chr17	22247196	C	G		90	8	0,08163					**0/2**						

chr17	22252187	T	G		257	30	0,10453	**0/5**	**0/1**	**5/14**	**4/9**	**4/15**	**2/4**	**0/1**	**0/6**	**0/1**	**2/9**	**0/7**
chr17	22252233	T	C		178	57	0,24255	**2/6**	1/4	14/14	7/12	**8/14**	**2/7**	1/1	**3/8**	**1/2**	5/11	**3/7**
chr17	22253208	T	A		1257	68	0,05132	**0/1**	**0/1**		**0/1**	**0/1**	0/1		0/2	0/1		
chr17	22253258	T	C		824	100	0,10823				**1/1**	**0/2**	**0/1**		**0/1**			
chr17	22261394	C	T		85	10	0,10526											
chr17	32366414	C	G		15	7	0,31818	**5/5**	**3/3**	**7/8**	**6/6**	5/13	**6/6**	1/3	0/1	**7/7**	**6/8**	**4/4**
chr17	32366781	C	G		23	11	0,32353	**6/6**	**11/14**	**8/11**	**9/10**	**5/9**	**9/10**	0/2	3/5	**9/9**	**9/9**	**9/9**
chr17	78071164	G	C	CCDC40	117	22	0,15827	0/5	0/2	0/4	1/3	2/7	1/6		1/8	3/3	0/7	1/7
chr17	78092383	G	A		146	15	0,09317		0/10		0/2	0/17					0/1	0/1
chr18	7231974	C	T	LRRC30	113	7	0,05833	0/17	0/18	0/6	0/20	0/3	0/1	0/1	1/14	0/20	0/1	
chr18	57638343	C	T		18	6	0,25	**2/4**	**3/3**	2/2	**1/1**	0/2	0/3	0/5	0/8	0/5	**0/4**	
chr18	70423240	T	A		83	8	0,08791	1/7	0/3	0/6	2/4	0/5	3/7	0/2	3/8	0/4	3/8	2/3
chr19	4513189	C	G	PLIN4	68	20	0,22727		0/20	1/1	0/7							
chr19	5685262	C	T		46	19	0,29231		0/1	**2/2**	**3/3**	3/4					0/2	
chr19	32655494	A	G		7	3	0,3					0/2			0/1		0/1	
chr19	49442737	A	G	DHDH	19	7	0,26923	0/15	2/11	**3/5**	3/5	0/10	**12/17**	2/4	**6/6**	**5/7**	**3/12**	0/4
chr19	52793612	A	G	ZNF766	131	47	0,26404	**5/16**	**6/6**	**2/2**	**4/10**	**6/25**	1/11	0/10	**14/14**	**15/34**	**10/21**	1/2
chr20	3674197	G	T	SIGLEC1	71	14	0,16471	**20/27**	**16/76**	0/15	0/58	**17/82**	**17/46**	0/4	0/21	**13/70**	**6/67**	**10/79**
chr20	25261002	C	G	PYGB	149	33	0,18132	**8/25**	8/30	2/8	2/28	0/13	0/7	1/4	**20/27**	**10/15**	4/19	**9/10**
chr20	32441208	G	T		73	18	0,1978			0/15	**4/4**				0/1			
chr20	39801342	G	A		78	17	0,17895				0/19	0/30			24/25	1/1	1/1	
chr20	47268008	T	G	PREX1	83	7	0,07778	**3/13**	1/14	**4/8**	**7/21**	**34/52**	0/11	1/2	**10/56**	0/6	2/10	0/3
chr20	62325612	G	T		56	22	0,28205				**3/4**					0/2		
chr21	11181412	G	C		22	8	0,26667											
chr21	37771809	C	G		33	49	0,59756	**7/7**	**9/9**	**8/8**	**10/10**	**15/15**	**10/10**	0/1	**11/11**	**7/7**		**2/2**
chr22	39496311	C	T	APOBEC3H	55	10	0,15385											
chrX	61731105	C	G		116	13	0,10078	**0/10**	**0/2**	**0/8**	**1/31**	**0/12**	**1/33**		0/8	**0/9**	**0/24**	**0/14**
chrX	102339379	C	T	NXF3	70	22	0,23913								0/1	0/1		0/1
chrX	123695668	C	T		78	9	0,10345	2/13	**3/8**	0/2	**6/11**		2/3	**4/6**	**6/6**	**13/22**		

chrom: chromosome
pos: position of the chromosome
ref: reference allele
alt: alternative allele
VAF: variant allele frequency in the bulk sample

Supplementary Table 5. Somatic SNVs identified in the CTC samples from patient with ER+ tumor (#3).

chrom	pos	ref	alt	gene	Nref	Nalt	VAF	1 CTCa	1 CTCb	1 CTCc
chr1	11132229	C	A		30	9	0,23077	0/4	0/3	0/3
chr1	24435237	G	C		46	7	0,13208	0/4	1/1	
chr1	36643441	C	T		94	23	0,19658			
chr1	52824810	C	A	CC2D1B	78	25	0,24272			
chr1	76346910	T	C		19	5	0,20833	0/24	0/17	0/22
chr1	111984006	C	A	WDR77	85	27	0,24107	0/38	0/39	**30/50**
chr1	114246799	G	A		88	16	0,15385	0/7	0/4	0/6
chr1	114308877	G	C	RSBN1	213	36	0,14458			
chr1	142535606	G	C		11	4	0,26667	3/46	1/21	2/24
chr1	145522692	C	T	PEX11B	69	5	0,06757	0/16	0/19	0/30
chr1	150938651	G	A	CERS2	49	7	0,125	0/65	0/136	0/6
chr1	152800232	C	T	LCE1A	18	4	0,18182		0/3	
chr1	158914744	C	T	PYHIN1	109	7	0,06034	0/4	0/6	0/14
chr1	160000321	G	C	PIGM	81	11	0,11957	0/21	0/29	**21/21**
chr1	165414185	C	G		48	5	0,09434	0/20	0/22	0/11
chr1	183532278	G	C		58	6	0,09375	0/31	0/36	0/11
chr1	183616845	C	A		126	12	0,08696	0/6	0/8	0/7
chr1	186008013	T	A	HMCN1	73	11	0,13095	0/7	0/8	0/12
chr1	201435171	G	A	PHLDA3	98	14	0,125	0/8	0/12	0/5
chr1	203316618	C	T	FMOD	82	11	0,11828		0/1	
chr1	218478451	C	T	RRP15	118	13	0,09924	0/29	0/32	0/23
chr1	220300113	G	A	IARS2	157	9	0,05422	0/21	0/14	0/14
chr1	225539393	G	C	DNAH14	81	5	0,05814	0/26	0/7	0/6
chr1	231004179	C	A	C1orf198	138	19	0,12102			
chr1	240370306	G	A	FMN2	109	6	0,05217	0/7	0/13	0/6
chr1	247267414	G	A	ZNF669	101	14	0,12174	0/18	0/11	0/4
chr1	248097233	C	G		189	62	0,24701	**3/11**	0/12	**4/6**
chr2	2094937	G	A		9	7	0,4375	0/1		
chr2	15514818	C	T	NBAS	84	77	0,47826	0/1	0/1	**7/7**
chr2	15614302	C	G	NBAS	34	60	0,6383	**6/7**	0/5	0/1
chr2	38301769	G	A	CYP1B1	32	31	0,49206			

chr2	64574771	G	A		12	6	0,33333	0/41	1/28	**12/12**
chr2	92305747	A	C		160	14	0,08046	0/1	0/5	0/3
chr2	112752676	G	A		119	11	0,08462			0/1
chr2	113993039	G	C	PAX8	32	8	0,2			
chr2	113993040	G	C	PAX8	33	8	0,19512			
chr2	120861681	G	A	EPB41L5	39	6	0,13333	0/17	0/18	0/11
chr2	128935345	G	C		123	14	0,10219	0/1	0/5	0/25
chr2	170518822	C	A	CCDC173	53	10	0,15873	0/1	0/4	0/10
chr2	173368888	G	A	ITGA6	71	6	0,07792	0/3	0/3	0/11
chr2	179469960	C	T	TTN	81	7	0,07955	0/2	0/4	**3/5**
chr2	179477465	G	A		45	9	0,16667	0/5	0/8	3/17
chr2	179497066	C	G	TTN	97	17	0,14912		0/1	0/1
chr2	179497145	C	G		72	12	0,14286		0/2	1/2
chr2	183847991	G	A		86	15	0,14851			
chr2	211069344	C	G	ACADL	124	15	0,10791	0/5	0/6	0/4
chr2	211503832	A	G		9	13	0,59091	**16/17**	0/35	**57/57**
chr2	216965052	C	T	TMEM169	112	18	0,13846	0/12	0/13	0/33
chr2	230705585	C	T	TRIP12	49	10	0,16949	0/3	0/3	0/5
chr2	232146696	G	A		47	6	0,11321	0/1	0/14	0/7
chr2	234663512	C	G	LOC100286922	113	17	0,13077	0/4	1/28	**10/31**
chr3	10858189	G	A	SLC6A11	7	11	0,61111			
chr3	44437898	G	A	TCAIM	23	7	0,23333		0/1	
chr3	47453171	G	C	PTPN23	48	5	0,09434			
chr3	52382946	G	A	DNAH1	32	12	0,27273	0/1	0/1	0/1
chr3	120424944	C	T	RABL3	112	25	0,18248			
chr3	140401869	C	T	TRIM42	210	53	0,20152	0/15	0/21	**5/9**
chr3	150916551	C	A		72	14	0,16279	0/6	0/5	2/3
chr3	151105646	G	A	MED12L	23	4	0,14815	0/77	1/60	**27/115**
chr3	160474432	C	T	PPM1L	66	20	0,23256			
chr4	86844	G	C	ZNF595	104	13	0,11111	0/9	0/3	0/15
chr4	6862654	C	T	KIAA0232	157	23	0,12778	0/39	0/19	0/59
chr4	49107435	C	T		54	6	0,1	1/14	0/7	0/3
chr4	49121633	C	T		22	5	0,18519	0/1		0/2
chr4	85600189	C	T	WDFY3	86	8	0,08511	0/9	0/9	0/13
chr4	110384489	C	G	SEC24B	99	12	0,10811	0/35	0/21	0/11

chr4	119219842	G	A		52	7	0,11864	0/12	0/12	0/14
chr4	123141475	C	T		89	11	0,11	0/10	0/11	0/2
chr5	5464616	C	T	ICE1	68	10	0,12821	0/11	0/21	0/17
chr5	23527657	C	A	PRDM9	145	8	0,05229		0/1	
chr5	34914888	C	T	RAD1	165	18	0,09836	0/95	0/165	**50/163**
chr5	35873622	G	C	IL7R	186	18	0,08824	0/3	0/10	0/2
chr5	49706754	G	C	EMB	158	19	0,10734			
chr5	54566500	C	A	DHX29	60	8	0,11765	0/13	0/74	0/22
chr5	72185600	T	G		39	29	0,42647			
chr5	79783990	C	T		27	7	0,20588		0/3	
chr5	90085564	G	A	ADGRV1	38	8	0,17391	0/2	0/7	0/4
chr5	93856592	C	T	KIAA0825	103	15	0,12712	0/18	0/54	0/62
chr5	108387027	C	T		36	19	0,34545	0/18	4/24	**27/42**
chr5	127641162	C	G		105	7	0,0625	0/19	0/10	0/10
chr5	131892410	C	T		63	9	0,125	0/17	0/156	0/50
chr5	135186013	C	T		40	8	0,16667	1/2	0/10	0/1
chr5	140362154	G	C		48	5	0,09434	0/24	0/35	0/26
chr5	150487959	G	A		71	6	0,07792	0/15	0/19	0/15
chr5	153026479	C	T		88	10	0,10204	0/54	0/85	0/38
chr5	175828728	G	T		183	13	0,06633	0/1	0/1	
chr5	180046488	G	A	FLT4	51	6	0,10526			
chr6	25106600	G	A	CMAHP	141	13	0,08442	0/14	0/15	0/13
chr6	27420746	G	A	ZNF184	100	15	0,13043	0/79	0/44	**10/37**
chr6	31911715	G	A	CFB	37	6	0,13953	0/7	0/6	0/13
chr6	32947953	G	A		39	8	0,17021	0/25	0/24	0/50
chr6	33255271	G	C	WDR46	73	8	0,09877	0/2	0/26	0/30
chr6	33365791	C	T		57	6	0,09524			
chr6	43266213	C	G	SLC22A7	74	10	0,11905	0/19	0/15	**3/12**
chr6	44136845	T	A		12	17	0,58621			
chr6	51524756	C	T	PKHD1	94	10	0,09615	0/7	0/2	1/7
chr6	56357069	C	T	DST	262	28	0,09655	0/10	0/3	0/8
chr6	90605371	C	T	GJA10	50	12	0,19355	0/4		**20/24**
chr6	117709171	C	A	ROS1	61	19	0,2375	0/9	0/3	1/16
chr6	132966591	G	C	TAAR1	90	18	0,16667	0/3	0/2	0/5
chr6	134210631	G	A	TCF21	54	10	0,15625	0/32		0/14

chr6	143082665	C	T	HIVEP2	102	25	0,19685	0/1	0/1	**4/4**
chr6	158476010	T	C	SYNJ2	10	15	0,6	**8/8**	1/1	**4/4**
chr7	31378807	C	A		268	25	0,08532	0/10	0/2	0/1
chr7	37243705	G	A		39	11	0,22	0/9	0/11	**7/19**
chr7	45724706	G	C		45	7	0,13462	0/23	2/19	3/13
chr7	48092288	C	G		63	5	0,07353		0/1	0/15
chr7	73770862	C	A	CLIP2	92	12	0,11538	0/29	0/32	**15/52**
chr7	78130917	C	G	MAGI2	90	8	0,08163	0/56	0/27	0/107
chr7	98608879	G	C		120	21	0,14894	0/6	0/18	0/6
chr7	105903930	C	T	NAMPT	106	14	0,11667			
chr7	128355483	A	T		29	23	0,44231	**10/13**	**30/30**	**12/12**
chr7	142198021	G	A		29	20	0,40816	4/6	2/2	2/3
chr7	143000879	G	C	CASP2	68	13	0,16049	0/15	0/15	0/13
chr7	150918792	G	T		47	13	0,21667	0/1	0/3	
chr8	17406321	G	C	SLC7A2	98	23	0,19008	0/6	0/12	0/1
chr8	70674185	C	T		80	11	0,12088	0/12	0/11	3/11
chr8	71128985	A	T		156	12	0,07143	0/15	0/20	0/25
chr8	94807750	G	C		123	29	0,19079	0/1	0/10	**6/11**
chr8	125072480	C	G	FER1L6	146	13	0,08176	0/90	0/73	0/52
chr8	126079879	G	C	WASHC5	205	25	0,1087	0/12	0/10	1/19
chr8	139614387	G	T	COL22A1	94	21	0,18261	0/13	0/6	**13/48**
chr9	396908	G	A	DOCK8	93	21	0,18421	0/2	0/3	0/3
chr9	68416460	A	G		6	3	0,33333	0/1		
chr9	136645033	G	A		34	5	0,12821	0/2	0/3	**5/10**
chr10	24833280	C	T	KIAA1217	127	13	0,09286	0/21	0/19	0/5
chr10	29137288	C	T		56	7	0,11111	0/60	0/34	0/64
chr10	42364809	C	T		217	13	0,05652	0/1	0/2	0/2
chr10	42380448	C	T		65	8	0,10959	0/8	0/8	0/4
chr10	42396703	T	C		163	10	0,0578	0/11	2/16	1/10
chr10	74684172	C	T	OIT3	146	14	0,0875	0/32	0/32	0/5
chr10	102746526	G	A	MRPL43	103	10	0,0885	0/57	0/54	0/101
chr10	107005362	C	T	SORCS3	60	5	0,07692	0/36	1/23	0/64
chr10	127668567	C	G		30	5	0,14286	0/1	0/2	2/3
chr10	135178224	C	T	ECHS1	145	23	0,1369	0/13	0/1	0/27
chr11	3838631	G	A	PGAP2	37	5	0,11905	0/2		

chr11	5631977	C	T		84	15	0,15152	0/71	0/94	0/4
chr11	10793128	G	A	CTR9	47	5	0,09615	0/54	0/39	0/27
chr11	20636270	C	G	SLC6A5	172	18	0,09474		0/5	0/1
chr11	27147281	G	C	BBOX1	81	19	0,19	0/25	0/4	0/15
chr11	36596180	G	C		80	7	0,08046	0/12	0/2	0/8
chr11	43904723	C	T	ALKBH3	67	10	0,12987	1/20	1/8	3/7
chr11	56258722	A	C	OR5M8	33	42	0,56	**4/4**	0/3	**6/7**
chr11	64004652	T	G		23	7	0,23333			
chr11	65733309	A	G		117	17	0,12687	0/18	0/26	0/8
chr11	66259309	C	G		110	19	0,14729	0/13	0/58	**4/21**
chr11	66617694	G	A	PC	110	9	0,07563			
chr11	67800843	G	A	NDUFS8	63	6	0,08696	0/1	0/3	
chr11	75062476	G	A		21	5	0,19231			
chr11	76918399	C	G	MYO7A	22	5	0,18519	0/20	0/13	**8/18**
chr11	94533202	G	C	AMOTL1	68	7	0,09333	0/11	0/10	0/6
chr11	111896326	C	G	DLAT	39	15	0,27778	0/6	0/9	0/9
chr11	122650373	G	A	UBASH3B	22	10	0,3125	0/20	0/2	0/21
chr12	6936114	G	A	GPR162	323	29	0,08239	0/37	0/37	0/24
chr12	7640684	C	T		86	10	0,10417	0/8	0/9	0/6
chr12	13717309	C	T	GRIN2B	127	16	0,11189	0/24	0/52	0/8
chr12	19242275	G	A		33	6	0,15385	0/22	0/33	0/11
chr12	40729017	C	G		26	4	0,13333	0/21	0/18	0/7
chr12	49167995	G	A		9	4	0,30769	**4/6**	0/6	0/4
chr12	49233800	G	A	DDX23	61	29	0,32222	0/5	0/6	
chr12	50152144	T	G		206	11	0,05069	0/14	0/2	0/1
chr12	54964126	C	T	PDE1B	47	5	0,09615	0/24	0/25	0/12
chr12	108589661	C	T	WSCD2	87	29	0,25	**7/26**	0/19	0/28
chr12	112130725	T	C		33	20	0,37736	**6/11**	**5/17**	1/3
chr12	122694392	C	T		141	8	0,05369		0/1	
chr12	130840137	G	C	PIWIL1	152	21	0,12139	0/13	0/19	0/17
chr13	19748306	G	T		24	8	0,25	0/2	0/1	2/6
chr13	25058831	C	T	PARP4	40	5	0,11111			
chr13	25072256	C	G	PARP4	36	10	0,21739	0/1	0/2	
chr13	25807657	G	A		105	18	0,14634		0/2	
chr13	32691564	G	C	FRY	49	10	0,16949	0/10	0/39	**47/57**

chr13	88329976	A	C		71	21	0,22826	0/3	0/2	0/2
chr13	94482637	G	A	GPC6	74	23	0,23711	0/12	0/20	0/23
chr13	110815876	G	T	COL4A1	34	9	0,2093	0/41		**23/52**
chr14	20295851	T	C	OR4N2	46	13	0,22034	**24/33**	**10/21**	**15/15**
chr14	20872106	G	A		73	14	0,16092	0/2	0/3	
chr14	24909683	C	T	SDR39U1	108	13	0,10744	0/21	0/14	0/15
chr14	45711647	G	A	MIS18BP1	53	8	0,13115	0/1	0/3	0/3
chr14	62550926	C	T	SYT16	23	6	0,2069	0/9	0/3	0/18
chr14	75469985	G	T	EIF2B2	53	10	0,15873	0/52	0/37	0/48
chr14	89747379	G	C		33	45	0,57692	**27/27**	**11/11**	**10/10**
chr14	91633669	G	A	DGLUCY	64	7	0,09859	0/21	0/36	0/47
chr14	102508957	G	A		74	14	0,15909	0/35	0/15	0/29
chr14	106993959	T	G		94	18	0,16071	0/9	0/2	0/7
chr14	106993964	A	G		94	18	0,16071	**0/9**	0/2	0/7
chr15	35376912	G	A		45	24	0,34783	**5/5**	0/17	**4/4**
chr15	40900959	A	G		22	7	0,24138		0/1	
chr15	76222325	C	G	FBXO22	64	13	0,16883	0/2	0/1	
chr15	82574861	C	T	SAXO2	27	10	0,27027	**5/10**	0/4	0/17
chr15	83296163	T	C		34	51	0,6	0/3	0/109	**26/32**
chr15	84722439	G	T		19	22	0,53659	**1/1**	0/5	
chr15	91565389	C	G	VPS33B	21	6	0,22222	0/4	0/22	0/20
chr16	4015968	C	G	ADCY9	158	11	0,06509	0/9	0/11	0/2
chr16	4016231	C	T	ADCY9	197	18	0,08372	0/7	0/12	0/6
chr16	4242282	C	T	SRL	92	12	0,11538	0/66	0/34	0/88
chr16	4242436	G	T	SRL	84	8	0,08696	0/34	0/23	0/50
chr16	4242589	C	G	SRL	210	20	0,08696	0/146	0/178	0/217
chr16	4242658	C	G	SRL	166	19	0,1027	0/165	0/235	0/256
chr16	4242706	G	T	SRL	153	20	0,11561	1/108	0/159	0/180
chr16	4385055	C	G		125	11	0,08088	0/13	0/19	**7/35**
chr16	4626585	G	A	C16orf96	147	10	0,06369	0/2	0/21	0/20
chr16	7102026	G	T		135	16	0,10596	0/27	0/8	0/16
chr16	12609513	C	G		96	9	0,08571	0/9	0/3	0/27
chr16	22128033	A	G		101	17	0,14407	0/30	0/5	**7/42**
chr16	22189526	G	A		85	34	0,28571			
chr16	23640680	C	T		74	9	0,10843	0/3	0/2	1/6

chr16	23721620	G	A		69	11	0,1375	0/14	0/7	0/37
chr16	31105781	C	T		113	12	0,096	0/7	0/13	0/21
chr16	33865245	T	G		19	5	0,20833	0/2	3/4	0/1
chr16	33866416	G	T		30	5	0,14286	**0/2**	0/1	4/6
chr16	46388833	C	G		89	8	0,08247	**0/3**	0/6	0/7
chr16	46417328	A	C		77	9	0,10465	**7/51**	**3/51**	**4/76**
chr16	49669973	A	C	ZNF423	99	30	0,23256			
chr16	69703949	A	T	NFAT5	92	84	0,47727			0/1
chr16	71715801	G	A	PHLPP2	48	10	0,17241	0/16	0/12	0/11
chr16	89235159	C	T	LINC02138	21	12	0,36364	0/1	1/13	**6/8**
chr16	89831306	C	T	FANCA	83	11	0,11702	0/36	0/53	0/12
chr17	2202763	C	T	SMG6	50	36	0,4186	**5/5**		1/6
chr17	3477002	G	T	TRPV1	61	11	0,15278	0/9		0/12
chr17	4619304	C	T	ARRB2	177	38	0,17674			
chr17	7811758	A	C	CHD3	45	5	0,1	0/40	1/35	0/59
chr17	10370020	G	C	MYH4	45	4	0,08163	0/5	0/8	
chr17	18148904	C	G	FLII	94	16	0,14545			
chr17	25266585	C	T		10	4	0,28571	0/14	1/5	0/8
chr17	26370088	T	C	NLK	28	24	0,46154	**16/25**	1/57	**28/65**
chr17	28758746	C	G		40	7	0,14894			
chr17	36064949	G	C	HNF1B	75	10	0,11765	0/3	0/15	0/14
chr17	42148540	C	A		28	8	0,22222		0/3	1/5
chr17	57288086	G	C	SMG8	63	7	0,1	0/22	0/67	0/11
chr17	61957797	C	T	GH2	78	11	0,1236	0/5	0/31	0/14
chr17	67041339	A	T	ABCA9	126	8	0,0597	0/4	0/4	0/10
chr17	72700885	C	T	CD300LF	103	83	0,44624	**5/9**	2/9	**7/17**
chr17	73150472	G	A	JPT1	37	6	0,13953		0/1	
chr17	74465346	C	T	AANAT	103	14	0,11966	0/2		
chr18	10750123	G	A	PIEZO2	92	10	0,09804	0/12	0/9	0/29
chr18	14777917	G	A		37	6	0,13953		0/2	
chr18	56940306	C	T	RAX	40	10	0,2			
chr19	1923133	C	A	SCAMP4	80	29	0,26606		0/22	0/3
chr19	3719871	C	G		47	8	0,14545	0/2	0/2	3/3
chr19	4222643	C	T		57	12	0,17391	2/7	2/5	1/8
chr19	17597549	G	A	SLC27A1	91	9	0,09			

chr19	31770574	G	A	TSHZ3	53	8	0,13115	0/8	0/22	2/6
chr19	40884190	C	G		31	4	0,11429			
chr19	40886475	G	C	HIPK4	63	7	0,1			
chr19	40886994	G	T	HIPK4	62	17	0,21519	0/2		
chr19	41869472	A	T		37	9	0,19565	0/39	0/45	**46/77**
chr19	42891237	C	A		27	8	0,22857			
chr19	47960399	C	T	SLC8A2	9	6	0,4			
chr19	52873233	G	C		107	33	0,23571	0/8	0/3	**6/13**
chr19	52887745	C	G	ZNF880	64	7	0,09859	0/10	0/12	0/27
chr19	53056509	G	A	ZNF808	86	29	0,25217	0/13	0/10	0/30
chr19	53840451	T	A		62	12	0,16216	0/27	0/23	**13/51**
chr19	54668149	C	T		8	12	0,6	**26/45**	1/54	**46/68**
chr19	56692130	C	A		48	12	0,2	0/3	0/7	0/9
chr19	56736307	C	A	ZSCAN5A	45	6	0,11765	0/11	0/18	**15/20**
chr19	57088960	G	T	ZNF470	105	15	0,125	0/3	0/16	0/11
chr20	896930	C	G		15	11	0,42308	**11/15**	0/7	**8/14**
chr20	1161959	G	C	TMEM74B	143	15	0,09494		0/1	0/3
chr20	1285777	G	C	SNPH	53	6	0,10169	0/4	0/5	0/1
chr20	21689893	C	G	PAX1	78	7	0,08235			
chr20	31024121	G	T	ASXL1	101	12	0,10619	0/28	0/41	0/32
chr20	34130567	C	T		49	7	0,125	0/81	0/109	0/64
chr20	43243286	C	G	PKIG	81	11	0,11957	0/18	0/15	**9/21**
chr20	58381200	G	C	PHACTR3	108	14	0,11475	0/7	0/36	0/15
chr21	10920017	C	T		177	29	0,14078	1/14	0/23	0/14
chr21	33840085	C	G	EVA1C	101	10	0,09009	0/5	0/2	0/9
chr21	39325126	C	G		23	16	0,41026	**18/44**	0/14	2/16
chr22	17646245	C	T		53	10	0,15873	0/1	0/2	0/1
chrX	3533811	G	A		127	66	0,34197	**49/50**	0/40	**37/61**
chrX	17745920	G	A	NHS	187	16	0,07882	0/27	0/10	0/34
chrX	47001802	C	A	NDUFB11	88	14	0,13725	0/103	1/92	**14/26**
chrX	48382408	C	T	EBP	61	5	0,07576	0/36	0/34	0/49
chrX	48546836	C	T	WAS	25	23	0,47917		0/1	**4/4**
chrX	54952040	C	G	TRO	170	19	0,10053	0/2		
chrX	74387681	G	C		28	4	0,125	0/1		
chrX	74803561	G	T		20	4	0,16667	0/14	0/14	0/15

chrX	77913918	C	G		125	15	0,10714	0/12	0/10	0/24
chrX	103045413	C	G		126	18	0,125	0/18	0/24	0/60
chrX	105912555	T	A	CXorf57	31	31	0,5	**4/10**	2/5	2/15
chrX	111624264	C	T		57	7	0,10938	0/13	0/17	0/43
chrX	118123440	A	G	LONRF3	37	7	0,15909	0/11	0/8	**11/15**
chrX	123516468	G	C		57	8	0,12308	0/5	0/11	0/8
chrX	148048348	C	G	AFF2	149	15	0,09146	0/120	0/104	0/180
chrX	153587943	C	A	FLNA	76	25	0,24752		0/2	0/2
chrX	153599265	C	T	FLNA	94	12	0,11321			
chrX	153609602	A	T		19	8	0,2963			
chrX	153668752	C	T	GDI1	59	6	0,09231		0/2	0/1
chrX	154157976	C	G	F8	178	34	0,16038	0/30	0/36	0/34
chrX	154158408	C	G	F8	80	16	0,16667	0/2	0/3	0/6

chrom: chromosome
pos: position of the chromosome
ref: reference allele
alt: alternative allele
VAF: variant allele frequency in the bulk sample

Supplementary Table 6. Common, shared CTC and private CTC mutations for patient with TNBC (#1).

	**Non-duplicate read pairs**	**Mutations**	**Mutations present in bulk**	**Mutations present in other CTCs**	**Private mutations**
**15 WBCs**	24108408	37	0	9	28
**1 CTCa**	27107890	855	11	20	824
**1 CTCb**	24559245	631	16	26	589
**1 CTCc**	26994739	390	17	21	352
**1 CTCd**	22636812	654	14	17	623
**5 CTCa**	25402660	312	13	17	282
**5 CTCb**	25358787	309	18	25	266
**5 CTCc**	10696284	141	10	12	119

Supplementary Table 7. Common, shared CTC and private CTC mutations for patient with HER2+ tumor (#2).

	Non-duplicate read pairs	Mutations	Mutations present in bulk	Mutations present in other CTCs	Private mutations
**10 WBCs**	25537426	78	0	10	68
**1 CTCa**	19173148	1513	27	29	1457
**10 CTCa**	22432107	1661	18	36	1607
**1 CTCb**	17115512	1610	23	30	1557
**10 CTCb**	22961019	947	26	28	893
**1 CTCc**	27816697	2445	24	23	2398
**10 CTCc**	28769658	433	19	24	390
**10 CTCd**	6030197	460	10	12	438
**10 CTCe**	18428610	1483	19	37	1427
**1 CTCd**	13153192	1830	23	38	1769
**10 CTCf**	22380988	329	19	34	276
**10 CTCg**	12733448	421	15	34	372

Supplementary Table 8. Common, shared CTC and private CTC mutations for patient with ER+ tumor (#3).

	Non-duplicate read pairs	Mutations	Mutations present in bulk	Mutations present in other CTCs	Private mutations
**20 WBC**	30798109	89	0	10	79
**1 CTCa**	23949238	1032	23	16	993
**1 CTCb**	22602049	1614	4	22	1588
**1 CTCc**	24654506	1215	47	17	1151

Supplementary Table 9. Coverage of the exome sequencing of the primary tumor and PBMC samples.

**Patient ID**	**BC type**	**Coverage tumor**	**Coverage normal**	**Purity (ABSOLUTE)**
#1	TNBC	142,04	106,11	0,217185816
#2	ER-/HER2+	133,47	110,65	0,344594979
#3	ER+/HER2-	150,45	144,07	0,324138482
